# A comprehensive study on characterization of biosynthesized copper-oxide nanoparticles, their capabilities as anticancer and antibacterial agents, and predicting optimal docking poses into the cavity of *S. aureus* DHFR

**DOI:** 10.1371/journal.pone.0319791

**Published:** 2025-04-01

**Authors:** Ehab S. Gad, Salem S. Salem, Samy Selim, Mohammed S. Almuhayawi, Mohammed H. Alruhaili, Soad K. Al Jaouni, Amna A. Saddiq, Medhat E. Owda

**Affiliations:** 1 Department of Chemistry, College of Sciences, Jouf University, Sakaka, Saudi Arabia; 2 Botany and Microbiology Department, Faculty of Science, Al-Azhar University, Cairo, Egypt; 3 Department of Clinical Laboratory Sciences, College of Applied Medical Sciences, Jouf University, Sakaka, Saudi Arabia; 4 Department of Clinical Microbiology and Immunology, Faculty of Medicine, King Abdulaziz University, Jeddah, Saudi Arabia; 5 Special Infectious Agents Unit, King Fahad Medical Research Center, King AbdulAziz University, Jeddah, Saudi Arabia; 6 Department of Hematology/Oncology and Yousef Abdulatif Jameel Scientific Chair of Prophetic Medicine Application, Faculty of Medicine, King Abdulaziz University, Jeddah, Saudi Arabia; 7 Department of Biological Sciences, Faculty of Science, University of Jeddah, Jeddah, Saudi Arabia; 8 Chemistry Department, Faculty of Science, Al-Azhar University, Cairo, Egypt; SRM Institute of Science and Technology, INDIA

## Abstract

The eco-friendly method of producing copperـoxide nanoparticles through the use of okra fruit extract is a simple, economical, rapid, and sustainable technique. The resultant copperـoxide nanoparticles (CuO NP) were analyzed with several analytical methods, such as UV-vis spectroscopy, FourierـTransform Infrared Spectroscopy (FT-IR), and X-Ray Diffraction (XRD), Zeta potential, TransmissionـElectron Microscopy (TEM) and EnergyـDispersive X-ray (EDX) analysis. The CuO NP exhibited a maximum absorbance at 381 nm. The formation of CuO NP was further confirmed by characteristic bands observed at 534 and 588 cm^-1^. The monoclinic structure of the CuO NP was identified with prominent peaks detected at 2θ values of 32.47°, 35.43°, 38.64°, 48.68°, 53.38°, 58.14°, 61.39°, 66.11°, 67.82°, 72.27°, and 74.96°. The overall findings indicate that the nanoparticles had an average diameter in the approximate range of 10 to 30 nm based on the TEM analysis. The cytotoxicity study, conducted on Human Fibroblast normal HFB4 cell lines, indicated that the halfـmaximal inhibitory concentration (IC50) dose was 236.34 μg/mL. An IC50 dose of 109.46 μg/mL was found in antitumor effect studies using breast adenocarcinoma Mcf- 7 cell lines, revealing a good level of safety for CuO NP. According to the antibacterial study, *Staphylococcus aureus* and *Bacillus cereus* had inhibition zone diameters (IZDs) of 29.5 ± 0.7 mm and 24.6 ± 1.2 mm, respectively, making them the most vulnerable bacteria to CuO NP. In contrast, *P. aeruginosa* was the least sensitive strain, with a minimum IZD of 15 ± 1.6 mm. Compared to gram-negative infections, the CuO NPs were found to have a significantly higher antibacterial effectiveness versus Gram -positive pathogens. Molecular docking against dihydrofolate reductase (DHFR) of *Staphylococcus aureus* (PDB ID: 6P9Z) illustrated that the CuO NP was partially interlocked with the active site of 6P9Z by the fitting energy value of -44.93 kcal/mol through five classical hydrogen bonds with Ala7, Gln9, Thr46, Ser49, and Phe92. The last one is also generated by the marketing antifolate agent methotrexate (MTX), adding some MTX-like character to the CuO NP inhibitor.

## Introduction

In recent decades, nanotechnology has revolutionized material science, particularly through the use of nanoparticle-based techniques [1,2]. Unlike their bulk counterparts, nanoparticles exhibit significantly altered chemical, physical and biological properties, which has achieved the route for advancements in various fields. These advancements include the synthesis of nanoparticles for diverse applications such as bioimaging, carbon nanotubes, cancer therapy, drug delivery, environmental remediation, antimicrobial treatments and various scientific fields [3–10].

Nanotechnology, a rapidly growing field, focuses on the design and fabrication of nanoparticles with size from 1 to 100 nanometers. Recently, the formation of metal and metal oxide nanoparticles (NPs) has garnered widespread interest within the global scientific community [11–14]. These nanoparticles are extensively employed in sectors such as pharmaceuticals, medicine, agriculture, textiles, sensors, electronics, optical fibers, bio-labeling, consumer goods manufacturing, and antimicrobial research [15–21]

Various methods are available for synthesizing nanoparticles, including biological, physical, and chemical approaches [22]. Examples of these methods include hydrothermal processes precipitation, ultrasonic irradiation, chemical reduction, physical vapor deposition, microemulsion, plasma, and sol-gel techniques [23]. Among these, biological or “green” synthesis has emerged as a superior alternative to traditional physical and chemical methods, primarily due to its lower energy requirements, reduced cost, minimal environmental impact, and ability to produce biocompatible and stable nanoparticles [24]. Furthermore, the biological approach offers better control over nanoparticle morphology such as nanoporous, nanorods structures, nanospheres, and nanowires which is essential for different creative applications [25]. Plants, in particular, have been used to synthesize NPs of gold, silver, silicon, titanium, zinc, copper, palladium, and magnetite [26–29].

Among these, copper nanoparticles stand out for their low cost and high yield, making them ideal for environmental and biomedical applications [30]. Researchers have shown great interest in nanomaterials made from copper and copper oxide because of their distinctive characteristics, which make them valuable for various applications such in sensors [31], catalysts [32], optics [33], solar cells [34], environmental remediation [35], antimicrobial treatments [36], and more. However, traditional methods for synthesizing of metal oxide NPs, such as CuONPs, often involve harmful chemicals and harsh physical conditions, leading to the generation of toxic byproducts and environmental pollution [37].

To address these challenges, researchers have turned to plant-mediated synthesis as eco-friendly and sustainable alternative for producing CuO NP [38,39]. This green method harnesses the reducing and capping abilities of phytochemicals found in plant extracts, eliminating the need for toxic chemicals and energy-intensive processes. Several studies have successfully synthesized CuO NP using various plants (Table 1). One plant species explored for this purpose is Psidium guajava (guava), whose leaf extract has been shown to function as both a reducing and capping agent, enabling the creation of copper oxide nanoparticles with a distinct monoclinic structure. Similarly, the extract of Hibiscus rosa-sinensis has also been employed for the bioـsynthesis of CuO NP, highlighting the versatility of plant-based approaches [38,39].

**Table 1:** Some reported CuO NP synthesized from different plant sources.

Plant name	Precursor	Morphology & average size	Applications	References
*Ficus elastica*	CuSO_4_ ⋅ 5H_2_O	flower like 20 nm and 107 nm	Detection of Cd^2+^, Hg^2+^, and Pb^2+^	[40]
*Giant cane*	Cu(NO_3_)_2_ ⋅ 3H_2_O	Aggregated structure	Electrochemical sensor and Photocatalysis	[41]
*Rumex nepalensis* Spreng	Cu(NO_3_)_2_	Spherical shape 21 nm and 97 nm	Antioxidant and antibacterial activity	[42]
*Parthenium hysterophorus*	CuSO_4_	Spherical shape 59.99 nm	Rifampicin antibiotic degradation	[43]
*Aegle marmelos*	CuSO_4_	Different shapes 32 nm and	Photocatalytic and ntimicrobial activities	[44]
Okra fruit extract	CuSO_4_	Spherical and hexagonal shapes 10–30 nm	Anticancer, Antimicrobial activities, Molecular doking	Present study

Additionally, okra (*Abelmoschus esculentus*) fruit is rich in phytochemicals such as polysaccharides, flavonoids, and tannins which can serve as stabilizing and reducing agents in the synthesis of metal oxide NPs [37,45]. The current study focuses on synthesizing CuO NP using okra fruit extract through the green route. The synthesized NPs are then characterized for their shape, size, and optical properties using techniques such as UV-vis spectroscopy, FT-IR, XRD, Zeta potential, TEM-EDAX, and. Furthermore, this study emphasizes the antimicrobial, cytotoxicity and docking properties of the CuO NP, demonstrating their potential in these applications.

## Materials and methods

### Preparation of okra fruit extract

Okra fruits were washed, dehydrated, and sliced before being immersed in distilled water. The mixture was subjected to constant stirring at 50°C for 4 hours to facilitate complete extraction. The resulting solution was then centrifuged to separate the solid residue from the liquid extract. The liquid extract was subsequently filtered and refrigerated for future use.

### Green synthesis of CuO NP

Twenty five (25) milliliters of okra fruit extract were gradually added to 50 milliliters of 1mM copper sulphate solution under constant stirring at ambient temperature. The mixture was then heated at 70°C for four hours, inducing the creation of CuO NP. These NPs were isolated through centrifugation, subsequently washed with distilled water and methanol to eliminate any impurities. A final calcination step at 500°C for four hours yielded pure CuO NP.

### Characterization of CuO NPs

The CuO NP produced via biological synthesis were thoroughly analyzed using multiple analytical methods. To examine the optical characteristics of the nanoparticles, we employed a Shimadzu UV-1700 spectrophotometer for UV-visible spectroscopy, at a wavelengthـrange of 200 to 800 nm. The many groups of functions in charge of the CuO NP’ reduction, covering, and stabilisation, were identified by F-TIR was conducted on an Agilent Cary 630 FT-IR spectrometer. The samples for FTIR analysis were prepared using potassium bromide as a matrix. The structural of the CuO NP was identified through XRD analysis, utilizing a Seifert3003TT diffractometer equipped with Cu Kα radiation. For detailed examination of the nanoparticles’ morphology and EDX, TEM was employed using a JEOL (GEM ــ1010) device. A Zeta ـSizer was utilised to test the created CuONP’ zeta potential.

### CuO NPs cytotoxicity and application as antitumor agent

Cell lines of breast cancer McFـ7 and fibroblast normal HFB4 were used to test the cytotoxicity and antitumor properties of CuO NP at Science Way Company in Cairo, Egypt. To form a full monoـlayer ـsheet, 100 µl/well (105 cells/ml) was added to the 96 ـwell cultureـplate, and it was then incubated for 24 hrs at 37 °C. The growth material was then removed from the 96-well micro titerـ plates, and the cell layer was twice washed using fresh medium. CuO NPs were categorised into twofoldـ dilutions in RP-MI medium, and the layer’s physical characteristics, such as rounding and shrinkage, were evaluated in multiple wells. Once the mixture was prepared, 20 microlitres of MTT mixture were added to each hole. For five minutes, media were placed on a table that is trembling and shaken at 150 rpm in order to completely integrate the MTT. For four hours, cultivate at 37°C with five% CO2 to enable the MTT to metabolise. The formazan was well mixed with the metabolise, allowed to settle for 3-5min, and the mixture’s spectral density was established at 560 nm.

### Application of CuONPs as antibacterial agent

To assess the biosynthesised CuO NP’ antimicrobial qualities, four harmful bacteria were used. The following bacteria were cultivated on Mueller-Hinton agar media: *P. aeruginosa, B subtilis S aureus, and E. coli*. The inhibitory impact was examined using the well diffusion technique for seven distinct CuO NP dosages against the four infections. Medium was poured to petri plates after 0.5 mL of each organism’s inoculum had been introduced. Wells were prepared using a 7 mm diameter sterilised cork porer as a standard reference, and 0.1 mL of CuO NPs at different concentrations (4000- 62.5 μg mL^-1^ were then added. Every plate was incubated at 37°C for a full day. Each well’s zone of inhibition (IZD) was calculated utilising a ruler and expressed as IZD (mm).

### *In silico* molecular docking

**Fig 1:**
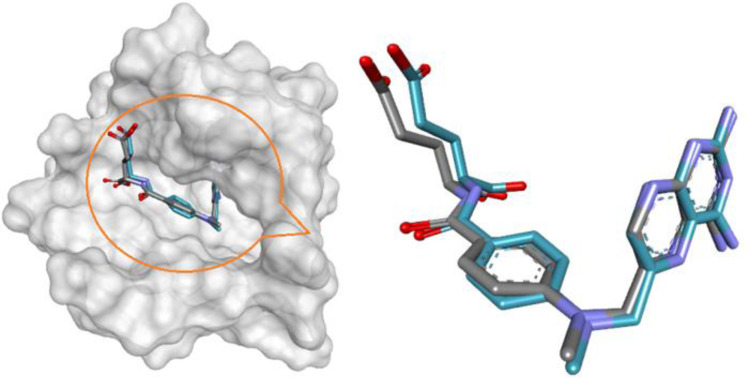
*i*GEMDOCK validation. Crystal MTX (cyan) and the docked one (grey) display similar bindingـorientation in the bindingـpocket of 6P9Z with RMSD 0.5822 Å.

According to the previously published protocol [46,47], a flexible-docking prediction was performed by *i* GEMDOCK app version: 2.1. The optimal docking posture of CuO NP into the cavity of S. aureus DHFR (PDB ID: 6P9Z), a significant biological target, was predicted using accurate docking based on a general evolutionary program (GA) and an empirical gaining model. The three-dimensional (3D) structure of 6P9Z complexed with methotrexate (MTX) was downloaded from RCSBــProtein DataــBank (https://www.rcsb.org) and in order to confirm that the docking approach worked, the co-crystallized blocker MTX was removed and re-docked to the appropriate catalytic domain (*i*GEMDOCK validation) (Fig 1). As demonstrated in Fig 1, the docked ligand (MTX) shows the Root MeanـSquare Distance (RMSD), which falls within the dependable range of 0.5822 Å, and shares the identical interaction type as the crystalline one, validating the efficiency of this protocol. Next, CuO NPs crystallographic -information file (CIF) was derived from the materials project website (https://legacy.materialsproject.org/) and modified by VESTA 3 software [48] then saved as pdb format and finally docked with 6P9Z.

## Results and discussion

### UV and Zeta potential analysis

**Fig 2:**
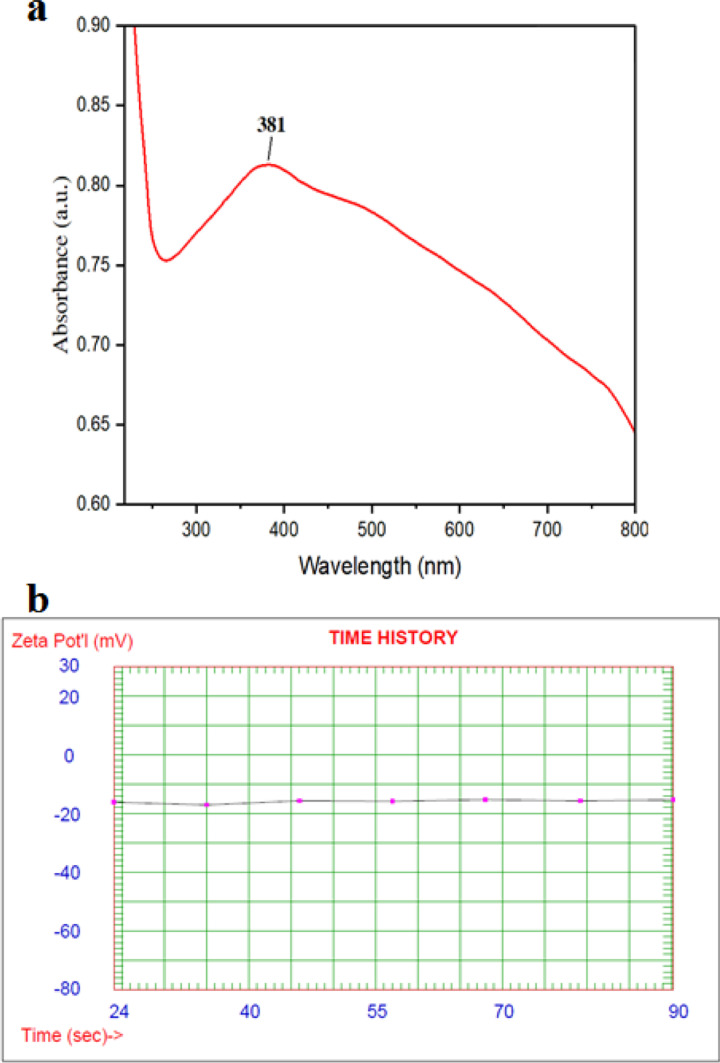
(a) UV-vis absorptionـspectrum and (b) Zeta potential of CuO NP.

A visible colour shift and UV-vis spectral analysis were used to demonstrate the formation of CuO NP. Fig 2a illustrates the absorption spectra, revealing a distinct peak at 381 nm. This observation falls within the expected absorption range of 300-400 nm for CuO NP, confirming their successful synthesis [49]. To appraise the stability of the synthesized nanomaterials, zeta potential measurements were conducted at pH 7. The zeta potential, which quantifies the surface charge density, is depicted in Fig 2b. Previous studies have reported varying zeta potential values for biosynthesized CuO NP, Kumari et al. observed a zeta potential of -34.12 mV for CuO NP synthesized using *Calotropis gigantea*. These nanoparticles demonstrated stability over a 72 hour period [50]. Muthuvel et al. reported a zeta potential of -27.12 mV for biosynthesized CuO NP, which was significantly higher than chemically synthesized counterparts (-3.14 mV). This suggests enhanced stability for the bio-based nanoparticles [51]. In our study, the biosynthesized CuO NP exhibited a zeta potential of -15.39 mV. This value is comparable to those reported for nano-CuO prepared from Neem and Jujube extracts [52]. indicating moderate stability. The biosynthesized CuO NP possess characteristic optical properties and a surface charge that contributes to their colloidal stability. The variation in zeta potential values across different studies highlights the influence of the biological source and creation conditions on the final properties of the NPs [52].

### FTIR spectroscopy

**Fig 3:**
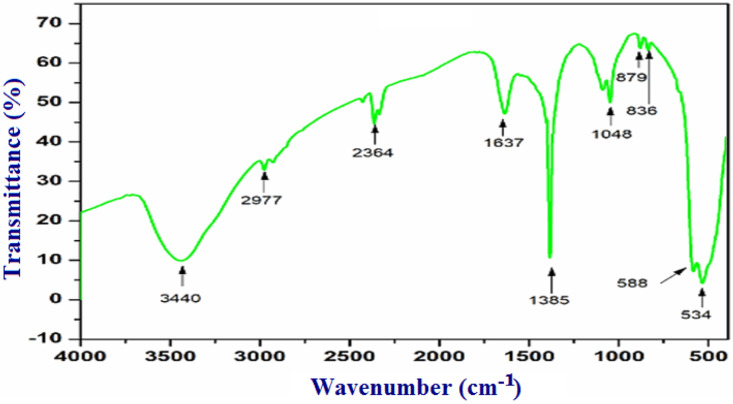
FTIR of the synthesized CuO NP.

The FTIR spectrum revealed distinct peaks at 3440, 2977, 2364, 1637, 1385, 1075, 879, 836, 588, and 534 cm^-1^. Fig 3 provided a detailed analysis of these absorption bands, the broad band at 3440 cm^-1^ attributed to O H stretching vibrations in hydroxyl groups. While the peak at 2977 cm^-1^ is attributed to C–H stretching vibrations of alkanes [53]. The pronounced peak at 2364 cm^-1^ suggests the presence of CO molecules that may have been absorbed during synthesis [54]. A sharp band at 1637 cm^-1^ is associated with C = C stretching vibrations, and Peaks at 1385 cm^-1^ and 1075 cm^-1^ are attributed to C = C stretching and O-C stretching of ester groups, respectively [55]. Bands below 1000 cm^-1^ are primarily related to metal-oxygen bonds, with Cu-O bonds appearing at 587 cm^-1^ and 534 cm^-1^. Notably, the absence of bands between 605 and 660 cm^-1^, characteristic of Cu-O (cuprous oxide), suggests that the synthesized CuO NPs are of high purity [56]. The observed vibrational bands in the IR spectrum are consistent with functional groups generated by ingredients that are biologically active, like alkaloids, flavonoids, proteins, carboxylic acids, and polyphenols. These compounds likely act as reducing and stabilizing agents in the preparation of CuO NP [57]. These results align with other reports on the creation of CuO NP using plant extracts [58,59]. Various studies have reported absorption peaks for CuO nanoparticles at different wavenumbers, including 615 cm^-1^ [60], 590 cm^-1^, 628 cm^-1^ [51], 624 cm^-1^ [61]. Additionally, some research has identified bands at 873 cm^-1^, 595 cm^-1^, 526 cm^-1^, and 475 cm^-1^, which are ascribed to the Cu–O group’s stretching oscillations in CuO NP [62,63].

### X-ray diffraction analysis

**Fig 4:**
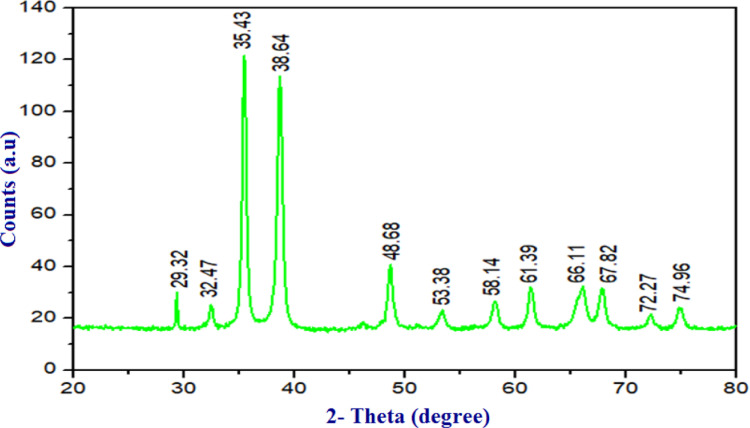
XRD of the green synthesized CuO NP.


The results presented in Fig 4, for XRD pattern revealed distinct, intense peaks at the following 2θ angles: 32.47°, 35.43°, 38.64°, 48.68°, 53.38°, 58.14°, 61.39°, 66.11°, 67.82°, 72.27°, and 74.96°, which correspond to the crystallographicـplanes (110), (002, (111, (202), (020, (202, (113), (311), (113), (311), and (222), respectively. The observed diffraction reflections closely match those in JCPDS Card No. 45-0937, which is characteristic of bulk CuO materials [64,65]. All identified diffraction peaks are consistent with a monoclinic crystal structure, and the production of CuO NP is highly pure, as seen by the absence of else peaks from different phases. Moreover, the sharp and well-defined nature of the CuO reflections in the XRD patterns indicates the well-crystalline character of the NPs. The average crystalliteـsize of the CuO NP was determined by Debye-Scherrer equation:

$$D=k\lambda /\left(\beta \;cos\;\theta \right)$$

where:

D =  crystalliteـsize, k =  0.89, λ =  X-ray Wavelength of, β =  (FWHM) fullـwidth at half maximum of the peak in radians

θ = Bragg diffraction angle

Using the FWHM of the most sharp peak, corresponding to the (002) diffraction plane, the crystallite size average was calculated to be 25.73 nm [66]. This calculation provides insight into the nanoscale dimensions of the synthesized CuO particles. Calcination is effective in producing crystalline nanoparticles with reduced contamination. The purity of the nanopowder was validated by the exclusive presence of the CuO NP XRD peak, confirming the absence of any impurities. The XRD analysis thus indicated the successful synthesis of pure, crystalline CuO NP with a monoclinic structure and offers a quantitative estimate of their average size. These findings are crucial for understanding the structural properties of the synthesized nanomaterials and their potential applications [67,68].

### Morphological analysis

#### TEM, SAED, and EDAX analysis.

**Fig 5:**
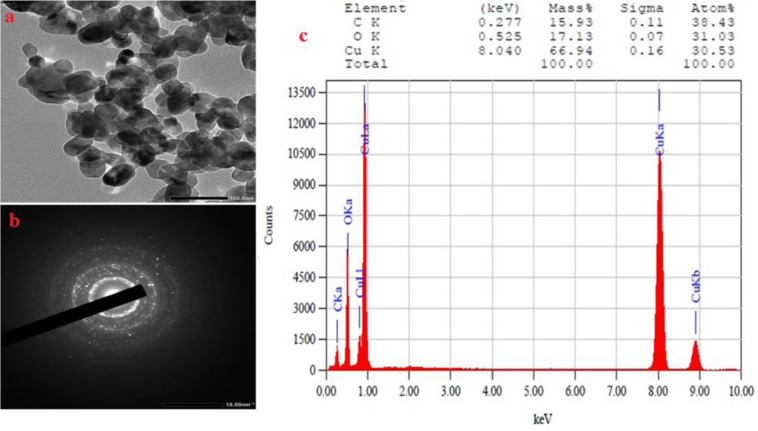
a) TEM image, b) SAED profile, and c) EDX of the green synthesized CuO NP.

As shown, Fig 5a revealed a diverse range of morphologies, predominantly Spherical and hexagonal, with average particle sizes from 10 30 nm. This size range is consistent with other reports on green synthesized CuO NP. Gu et al. (2022) formed the Cu NPs by *Calendula officinalis* extracts, reporting particles less than 100 nm [69]. Liyuan et al. (2022) produced Cu nanoparticles using *Alhagi maurorum* extract, with sizes ranging from 10 to 60 nm [70]. Chinnaiah et al. produced Cu nanoparticles (rather than CuO) using *Datura metel L.* extract, achieving an average diameter size of 19.56 nm [71]. Kumar et al. utilised leafs of *Andean sacha-inchi* through a heating process, to synthesize semicrystalline, well-dispersed Cu NPs resulting in particles of about 46 nm [72]. Murthy et al., effectively produced CuO NP using *Vernonia amygdalina Del.* extract, producing monoclinic structures with a mean particle size of 19.7 nm [73]. The SAED pattern displayed four distinct bright circular concentric rings (as illustrated in Fig 5b), attributed to the random orientation of crystal planes, corresponding to Debye–Scherrer diffraction rings, which confirm the crystalline nature of the CuNPs. Consequently, we observed that the particle size and SAED pattern derived from TEM observations align well with the results obtained from XRD analysis. This approach for the elemental composition analysis of nanomaterials, providing both quantitative and qualitative data. Chandrasekaran et al. identified metallic copper nanoparticles through EDAX analysis in their study involving the synthesis of CuNPs using the extrasct of *Beta vulgaris* [74]. Similarly, in the biosynthesised of CuO/C nanocomposites, the presence of oxygen, carbon, and copper was confirmed with their elemental composition also assessed via EDAX analysis [75]. Also, peaks corresponding to copper and oxygen were observed during the synthesis of CuO NP using extracts from *Cyperus rotundus* and Cynodon dactylon [76]. When fig leaves were used, the copper and oxide content was found to be 3.56% and 75.14%, respectively, while 21.31% of the plantـextracts included additional elements contaminants [77]. Our results are consistent with these findings, indicating a copper content of 66.94% and oxide contents of 17.13%, respectively, with other impurities at 15.93%.

#### Cytotoxicity and Antitumor popertie of CuO NP.

**Fig 6:**
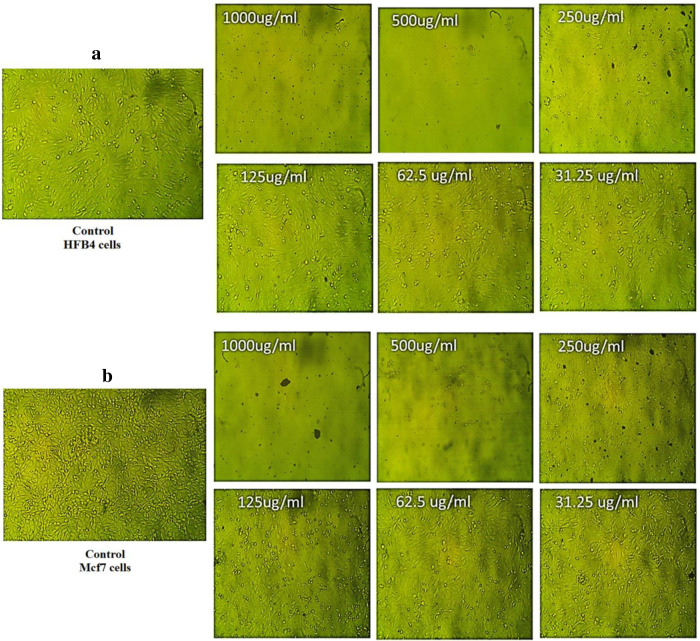
HFB4 (a) and Mcf-7 cell (b) after treated with CuO NP.

Testing biological materials for experimental cytotoxicity against types of normal cells is the initial stage of assessing their safety after being exposed to different concentrations of CuO NP, which were 500, 250, 125, 62.5, and 31.25μg/mL [78]. Fig 6a shows microscopic pictures of the HFB4 cell lines’ morphological alterations treated with 1000–250 μg ml-1 with CuO NP. Utilising the curve, IC50 values were calculated, which evaluate the pharmacological dosage concentration necessary to cause 50% of cells to die [74]. The IC50 value of the CuO NP produced on HFB4 cell lines was 236.34 μg/mL, according to the findings displayed in Fig 6 (S1 Table). CuO NPs were used in an anticancer research against Mcf-7 cell lines at several doses between 1000 and 31.25 μg/ml. Nanoparticles had an impact the Mcf-7 cell lines’ vitality, as seen in microscopic images. Based on the morphological changes observed in Mcf-7 cell lines exposed to 1000- 125 μg/ml of CuO NP, as shown in Fig 6b, it was believed that CuO NP had anticancer effect. CuO NP was 109.46 μg/mL, as seen in Fig 6b (S2 Table). CuO NP manufactured from F. religiosa were assessed by Sankar et al. using the MTT test against the A-549 cell line, achieving an IC50 value of 200 µg/ml [79]. Normal fibroblast (HuFb) cell line (IC50 value = 54.34 µg/mL), SKOV 3 cancer (IC50 value = 2.27 µg/mL), and AMJ 13 human breast cancer (IC50 value = 1.47 µg/mL) were the three cell lines against which the CuO NP that were synthesised from *Olea europaea* extract were tested for cytotoxic action [80]. Our findings lead us to conclude that there is clear and inexpensive proof of the anticancer potential of environmentally produced CuO NP.

**Fig 7:**
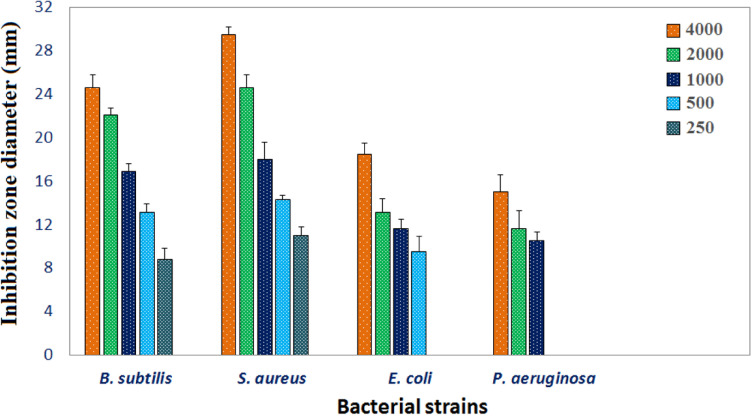
Antimicrobial activity for CuO NP.

#### Application of CuONPs as antibacterial agent.

The bactericidal efficacy of the biosynthesised CuO NPs was tested using four pathogens, two of which were gramme positive and the other two gramme negative. Bacteria that are most susceptible were *S aureus and B subtilis*, with 29.5 ± 0.7 mm and 24.6 ± 1.2 mm (IZD) respectively, based on data in Fig 7 (S3 Table). In contrast, *E. coli and P aeruginosa* growth were less affected by CuO NP, which had the smallest IZD and the lowest vulnerability of 18.5 ± 1 and 15 ± 1.6 mm respectively. The results demonstrated that CuO NP’ MIC against *B. subtilis* and *S aureus* microbial growth was superior to that of other strains, with MICs of 250µg/mL against both species. Conversely, *E. coli* and *P. aeruginosa* had MICs of 500 and 1000 µg/mL, respectively. Compared to gram-negative pathogens, CuO NP shown a substantially higher antibacterial impact against gram-positive pathogens since (Fig 7) abolished the antibacterial action of gram ـpositive infections. According to earlier studies, CuO NP interact with bacterial cell membranes to increase their antibacterial action [81]. This is because CuO NP have a large specific surface area [67]. CuO NP and the cell membrane have more possibilities to come into touch as a result, which encourages positive interactions with bacteria and boosts activity. CuO NP interact negatively with bacterial cell membranes, causing reactive oxygen species to be produced and the elements that make up the membrane of bacteria to be disrupted or oxidized [82]. Furthermore, these findings imply that, in contrast to Gram-negative bacteria, gram ـpositive bacteria are more vulnerable to CuO NP [82]. The two species of bacteria have different cell wall architectures, which explains this impact. The complex cell wall structure of gram-negative bacteria includes an outer membrane made of lipopolysaccharides, which acts as an extra barrier against antibacterial drugs [83]. Because of its negative charges, this outer membrane makes the cell wall thicker and prevents antibacterial substances from penetrating the cell, decreasing their efficacy. Gram-positive bacteria, on the other hand, are more susceptible to antibacterial drugs due to their simpler cell wall construction [84].

#### Molecular docking results of antibacterial target.

**Fig 8:**
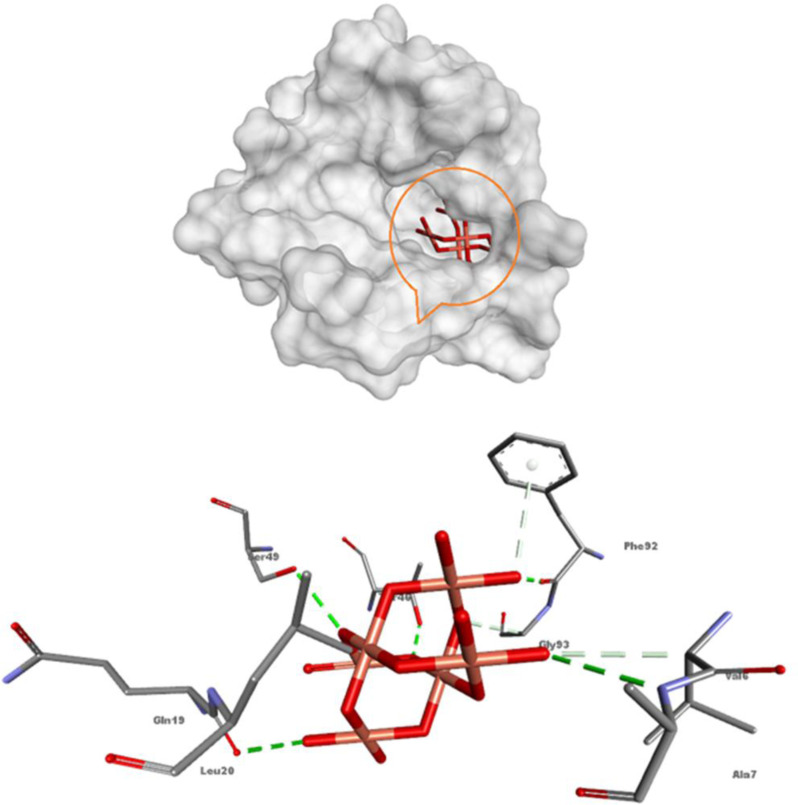
Molecular docking 3D representation of CuO NP with the active site of *S. aureus* DHFR (PDB ID: 6P9Z).

The results of the antibacterial screening indicate that the CuO NP had the greatest inhibitory effect on *Staphylococcus aureus* with an IZD of 29.5 ± 0.7 mm (Fig 7). To gain insights into their action mechanism, *in silico* molecular docking was conducted against DHFR of *S. aureus*, a promising target for antimicrobial agents [85]. A flexible docking simulation of CuO NP against 6P9Z (PDB ID) was implemented using the *i*GEMDOCK software version 2.1 [46]. As shown in Fig 8, CuO NP fitted into the active site of 6P9Z by the binding energy value of -44.93 kcal/mol through several intermolecular classical and non-classical hydrogen bonds. The classical H-bonds are generated between the CuO oxygen (as acceptors) and hydrogen donors NH and OH groups of amino acid residues, including Ala7, Gln9, Thr46, Ser49, and Phe92. Worthy to mention that the last one (Phe92, 2.06 Å) is also formed by interaction between the aromatic NH_2_ group of co-crystallized antifolate ligand MTX and Phe92 (2.91 Å), proving some MTX-like character to the CuO NP inhibitor [47]. Understanding protein–metal adhering and the network of molecules that results from the interaction of nanomaterials with biomolecules is crucial, and this understanding has inspired the development of molecular docking, a tool that can be used to predict the partnership affinity of protein–metal combinations as well as the obstacles and limitations that arise when binding is not possible [86]. In a prior work, AutoDock 4.2 was used to molecularly dock copper oxide against vital proteins of *L. monocytogenes* and *S. typhi*. The findings showed energies of binding against the proteins of *L. monocytogenes* and *S. typhi*, suggesting possible interactions and laying the groundwork for a deeper comprehension of the molecular underpinnings of the spotted antibacterial behaviour in vitro towards *L. monocytogenes* and *S. typhi* [87]. Molecular docking has a wide range of yet little-studied applications in the biological uses of CuO NP.

## Conclusion

The current study developed a low-cost, environmentally friendly method of producing bio-functionalized CuO NP using Okra fruit extract. As per the best of our awareness this is the novel work on the green fabrication bioinspired of CuO NP from Okra fruit extract and it also demonstrated the anticancer, antibacterial, and molecular doking properties of as-synthesized nanoparticles. Using FTIR, UV-Vis, XRD, EDX, and TEM examination, the form, architectural, and optical characteristics of the biogenic synthesised CuO NP have been investigated. The presence of a significant absorbance band at 381 nm and XRD peaks that matched crystalline monoclinic CuO NP perfectly validated the synthesis of CuO NP. The spherical and hexagonal particles, with a size range of 10–30 nm, were visible using the TEM method. The existence of solely Cu and O peaks in the EDX analysis verified the purity of the nanomaterial. The susceptibility of bacterial pathogens versus CuO NP was also spotted with MICs of 250 µg/mL for *B. subtilis* and *S. aureus*, while *P. aeruginosa* and *E. coli* were 1000 and 500 µg/mL, respectively. Nevertheless, their greatest anticancer effect was seen against Mcf- 7 and IC_50_ was 109.46 μg/mL. Molecular docking proved the ability of CuO NP to be a good inhibitor of DHFR; thus, it is considered an effective antibacterial agent against *S. aureus*. Thus, it will be subjected to further biological studies in our lab. Overall, the findings confirmed that CuO NP fabricated employing extract of Okra fruit have potential to be used as active agent in various biomedical uses after further detailed clinical investigations.

## Supporting information

S1 Table Viability and toxicity percent for normal HFB4 cells treated with different concentration of CuO NPs. (PDF)

S2 Table Viability and toxicity percent for cancer Mcf7 cells treated with different concentration of CuO NPs. (PDF)

S3 Table Antimicrobial activity of CuONPs. (PDF)
