# Supplementary material for: A comprehensive study on characterization of biosynthesized copper-oxide nanoparticles, their capabilities as anticancer and antibacterial agents, and predicting optimal docking poses into the cavity of S. aureus DHFR
Source: PLoS One. 2025 Apr 1;20(4):e0319791. doi: 10.1371/journal.pone.0319791 (PMC11960894; doi:10.1371/journal.pone.0319791)
Supplement: S1 Table — (PDF) [file pone.0319791.s001.pdf]

S1 Table: Viability and toxicity percent for normal HFB4 cells treated with different concentration of CuO NPs.

ID	ug/ml	O.D			Mean O.D	±SE	Viability %	Toxicity %	IC50 ± SD
HFB4	--------	0.72	0.724	0.716	0.72	0.002309	100	0	ug
CuONPs	1000	0.022	0.018	0.024	0.021333	0.001764	2.962962963	97.03703704	236.34 ± 2.37
	500	0.02	0.02	0.021	0.020333	0.000333	2.824074074	97.17592593	
	250	0.333	0.317	0.326	0.325333	0.004631	45.18518519	54.81481481	
	125	0.721	0.717	0.718	0.718667	0.001202	99.81481481	0.185185185	
	62.5	0.716	0.725	0.718	0.719667	0.002728	99.9537037	0.046296296	
	31.25	0.722	0.72	0.717	0.719667	0.001453	99.9537037	0.046296296	
