# Supplementary material for: A comprehensive study on characterization of biosynthesized copper-oxide nanoparticles, their capabilities as anticancer and antibacterial agents, and predicting optimal docking poses into the cavity of S. aureus DHFR
Source: PLoS One. 2025 Apr 1;20(4):e0319791. doi: 10.1371/journal.pone.0319791 (PMC11960894; doi:10.1371/journal.pone.0319791)
Supplement: S2 Table — (PDF) [file pone.0319791.s002.pdf]

S2 Table: Viability and toxicity percent for cancer Mcf7 cells treated with different concentration of CuO NPs.

ID	ug/ml	O.D			Mean O.D	±SE	Viability %	Toxicity %	IC50 ± SD
Mcf7	--------	0.753	0.751	0.761	0.755	0.003055	100	0	ug
CuONPs	1000	0.019	0.022	0.02	0.020333	0.000882	2.693156733	97.30684327	109.46 ± 1.52
	500	0.021	0.017	0.02	0.019333	0.001202	2.560706402	97.4392936	
	250	0.152	0.147	0.138	0.145667	0.004096	19.29359823	80.70640177	
	125	0.315	0.296	0.301	0.304	0.005686	40.26490066	59.73509934	
	62.5	0.6	0.588	0.613	0.600333	0.007219	79.51434879	20.48565121	
	31.25	0.731	0.726	0.728	0.728333	0.001453	96.46799117	3.5320088	
