# Supplementary material for: A comprehensive study on characterization of biosynthesized copper-oxide nanoparticles, their capabilities as anticancer and antibacterial agents, and predicting optimal docking poses into the cavity of S. aureus DHFR
Source: PLoS One. 2025 Apr 1;20(4):e0319791. doi: 10.1371/journal.pone.0319791 (PMC11960894; doi:10.1371/journal.pone.0319791)
Supplement: S3 Table — (PDF) [file pone.0319791.s003.pdf]

S3: Antimicrobial activity of CuONPs.

Bacterial strains	CuONPs (µg mL^{-1})	R1	R2	R3	Mean	Std
S. aureus	4000	29	29	30.5	29.5	0.7
	2000	26	23	25	24.6	1.2
	1000	20	18	16	18	1.6
	500	14	15	14	14.3	0.4
	250	11	12	10	11	0.8
	125	0	0	0	0	0
B. subtilis	4000	25	26	23	24.6	1.2
	2000	22	23	21.5	22.1	0.6
	1000	17	16	17.8	16.9	0.7
	500	13.5	14	12	13.1	0.8
	250	10	9	7.5	8.8	1
	125	0	0	0	0	0
E. coli	4000	18	20	17.5	18.5	1
	2000	15	12	12.5	13.1	1.3
	1000	13	10.8	11	11.6	0.9
	500	11	7.5	10	9.5	1.4
	250	0	0	0	0	0
	125	0	0	0	0	0
P. aeruginosa	4000	17	14	12	15	1.6
	2000	14	10	11	11.6	1.7
	1000	10.8	10	9	10.5	0.8
	500	0	0	0	0	0
	250	0	0	0	0	0
	125	0	0	0	0	0
